# *Insite*: Canada's landmark safe injecting program at risk

**DOI:** 10.1186/1477-7517-3-24

**Published:** 2006-08-09

**Authors:** Ernest Drucker

**Affiliations:** 1 Montefiore Medical Centre, 111 East 210 St., Bronx, New York City, 10467, USA

## Abstract

InSite is North Americas first supervised injection site and a landmark public heath initiative operating in Vancouver since 2003. The program is a vital component of that cities internationally recognized harm reduction approach to its serious problems with drugs, crime, homelessness and AIDS. InSite currently operates under a waiver of Federal rules that allow it to provide services as a research project. An extensive evaluation has produced very positive results for thousands of users. Normally such strong evidence documenting the successes of such a program, and the medical and public health significance of these positive outcomes, would be the basis for celebration and moves to expand the model and provide similar services elsewhere in Canada.

Instead, there is a distinct possibility that InSite will be closed by the newly elected Canadian Prime Minister Paul Harper – a conservative who has traveled to the US to visit George WQ Bush and come back antagonistic to harm reduction in all its forms. Because InSites federal waiver is expiring and up for renewal in September, the fear is that Mr. Harpers will not renew the approval and that the program will be forced to close down. The risks associated with the potential closure of InSite need to be fully understood. This editorial lays out these public health risks and the associated economic impact if InSite were to be closed.

In addition to preventable deaths and disease, InSites closure will cost Vancouver and British Columbia between $3.8 and $ 8.8 million in preventable health care expenses over the next two years.

## Background

*Insite *is North America's first supervised injection site – a landmark public heath initiative that has been operating since 2003 in Vancouver's Downtown Eastside. The program is a vital component of that cities internationally recognized harm reduction strategy addressing it's serious problems with drugs, crime, homelessness and AIDS. *Insite *serves more than 7,200 registered clients with 15,000 to 20,000 visits each month – all active intravenous users at the highest risk for HIV transmission and overdose. Insite has now been providing vital health care and referral services to drug users in Vancouver's downtown eastside for over two years and the results are impressive. (See Additional file 1)

InSite has been the subject of rigorous, independent research and evaluation since it opened its doors and the data collected so far are compelling in their implications. These studies convincingly demonstrate Insite's positive impact as a life saving public health program that does not increase drug use nor produce any adverse outcomes. As published in leading peer-reviewed journals,(see Additional file 2) these studies uniformly and persuasively find that the Insite program is producing important public heath and social outcomes and exceeding all of its targets.

InSite's three-year demonstration and research program provides the community, government, other health care agencies, and the global research community with much needed data about the efficacy of such a facility and its consequences both for health outcomes related to drug use and (of equal importance) for its local community's sense of safety and security. Much of that research has been conducted by investigators at the British Columbia Centre for Excellence in HIV/AIDS and published in leading peer-reviewed journals.

The principal research findings are that:

• *InSite *is leading to increased admissions to local detoxification programs and addiction treatment. [1]

• *InSite *has not led to an increase in drug-related crime, rates of arrest for drug trafficking, assaults and robbery were similar after the facility's opening, and rates of vehicle break-ins/theft declined significantly. [2]

• *InSite *has reduced the number of people injecting in public and the amount of injection-related litter in the downtown eastside. [3]

• *InSite *is attracting the highest-risk users – those more likely to be vulnerable to HIV infection and overdose, and who were contributing to problems of public drug use and unsafe syringe disposal. [4]

• *InSite *has reduced overall rates of needle sharing in the community, and among those who used the supervised injection site for some, most or all of their injections, 70% were less likely to report syringe sharing. [5]

• Nearly one-third of *InSite *users received information relating to safer injecting practices. Those who received help injecting from fellow injection drug users on the streets were more than twice as likely to have received safer injecting education at *InSite*. [6]

• *InSite *is not increasing rates of relapse among former drug users, nor is it a negative influence on those seeking to stop drug use. [7]

Since it came into being, *Insite *has reached over 7200 high risk injection drug users (usually those not otherwise in treatment) and decreased public injection, reduced needle sharing, prevented bacterial infections and overdose deaths; increased the use of withdrawal management (detoxification or tapering off drugs) and provided other addiction harm reduction and treatment services for over 4000 clients; while also increasing referral to other community resources that can help active drug users – e.g. mainstream medical, and mental health services. In addition *InSite *has not increased crime, public disorder, or drug dealing in its neighborhood, not led to increased relapse among former drug users, nor been a negative influence on those seeking to stop drug use altogether.

## The threat to close *InSite*

Normally such strong evidence documenting the many successes of such a pilot program, and the medical and public health significance of its very positive outcomes, would be the basis for celebration and lead to moves to expand the model and provide similar services elsewhere in Canada. Instead, there is a distinct possibility that *InSite *will be closed by the newly elected Canadian Prime Minister Paul Harper – a conservative who has traveled to the US to visit George W. Bush and come back to Ottawa hostile to harm reduction in all its forms – a sentiment that originates in Washington DC and appears to function as a loyalty test for international drug policies worldwide.

The InSite program operates under a waiver of Canadian Federal health rules that allow it to provide its services as a research project. Because InSites federal waiver is expiring in September, many fear that Mr. Harper will not renew its approval and that the program will be forced to close down. The risks associated with any potential closure of *InSite *need to be fully understood.

In addition to abandoning the care of so many of Vancouver's drug users and the needless pain and suffering this will inflict on them and their loved ones, it is possible to project a number of very specific consequences of any failure to continue it. By utilizing the data from the *InSite *studies that have been published in the peer review literature, we can predict a set of adverse outcomes and their related economic costs – all now prevented by *InSite*. These include:

*Failure to Prevent 22 overdose deaths*: based on a 5% mortality rate among 453 overdoses treated at *In Site*, if these had occurred in the community. [1]

*Failure to Prevent 112 hospitalizations for non – lethal overdose deaths*: based on a 25% hospitalization rate (including psychiatric) among 453 overdoses treated at *In Site*, if these overdoses occurred in the community. At a 2–5 day average length of stay for each such admission, this yields 224–560 extra hospital bed days and estimated costs (at $ 500/day) would total $112,000 to $280,000 [2]

*Failure to Prevent 2000 Emergency medical visits for injection mishaps*: Emergency room treatment of abscesses and other bacterial infections associated with unsafe injecting cost $1000 – $3000 per incident – annual savings $2 – 6, 000,000) [3]

*Failure to Prevent 100 Hospitalizations due to bacterial infections*: Based on 5% rate of hospitalization for the 2000 bacterial infections seen at Insite. Each such admission has an average length of stay of 15 – 20 days with a cost (at $500 per day) of $7500 – 15,000 per admission and a cost per year of $ 750,000 – 1 million [4]

*Failure to make 100 referrals to Methadone treatment*: with savings of $10,000/year/client in criminal justice and medical costs = $ 1 million [5]

The economic cost of closing InSite will far exceed the cost of operating it – by millions of dollars per year. The available data on the economic impact of *Insite *(in addition to the preventable deaths and disease that the programs closure will cause) indicate that the end of the program will cost Vancouver and British Columbia between $ 3,862,000 and $8,780,000 in additional health care expenses over the next two years. In addition to the increased demands that InSites closure would place on already scarce health care resources, *Insites *termination would also lead to thousands of additional arrests (which cost more than $10,000 apiece) court adjudications (another $10,000) and all the jail and probation time (not to mention public displeasure) associated with a return to public drug use by this large group.

As AIDS associated with injecting drug use continues to spread and ignites new regional epidemics of HIV – most dramatically evident today in the former Soviet Union – we need more *InSites*, not fewer ones. Vancouver's and Canada's struggle to develop and maintain innovative public health programs that more effectively deal with drugs and AIDS are ones we in the international public health community all share. But the end of *InSite *would be a setback not only for Vancouver and Canada – it would be a great loss for all of our efforts to build effective and sustainable public health initiatives for dealing with addiction and AIDS prevention everywhere in the world.

By granting renewal of *InSite's *federal approval, the Canadian federal government will be acting in the best interest not only of the citizens of Vancouver, but also in the best interests of all Canadians struggling to find effective responses to the nations daunting problems of AIDS and addictions. And, because of the high esteem in which Canadian public health is held worldwide, the actions of the Canadian government in regard to *InSite *will have a strong effect elsewhere. If negative they will embolden the opponents of harm reduction globally – reinforcing a dangerous trend to put some short term perceived political advantage ahead of public health evidence.

As the research on *InSite *makes so readily apparent, the failure to sustain InSite will most certainly condemn scores of Canadians to premature deaths (from drug overdose and AIDS) and thousands more to lives of increased disease, degradation, and criminal activity – all at inordinate public expense. The choice seems clear.

## Supplementary Material

Additional file 1 a brochure from Vancouver Coastal Health which gives an overview of the program and some examples of its work in the Downtown Eastside community. Click here for file

Additional file 2 a review which summarizes these research studies. Click here for file
