# Supplementary material for: Insite: Canada's landmark safe injecting program at risk
Source: Harm Reduct J. 2006 Aug 9;3:24. doi: 10.1186/1477-7517-3-24 (PMC1562403; doi:10.1186/1477-7517-3-24)
Supplement: Additional file 1 — a brochure from Vancouver Coastal Health which gives an overview of the program and some examples of its work in the Downtown Eastside community. [file 1477-7517-3-24-S1.pdf]

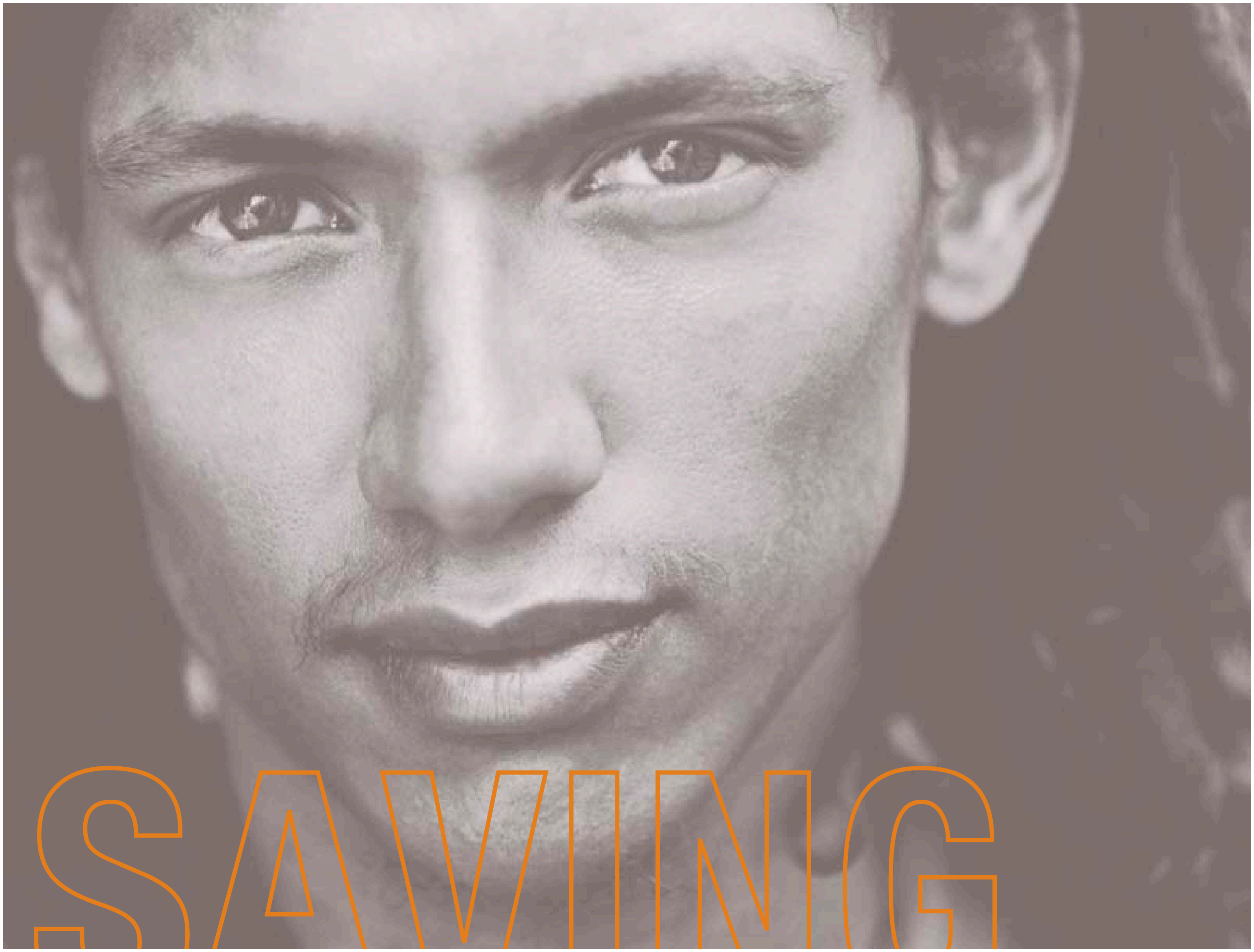

SAVING LIVES

Vancouver's Supervised Injection Site

Vancouver CoastalHealth
Promoting wellness. Ensuring care.

Identifying the Problem

Vancouver Coastal Health recognizes that people with addictions are some of the most vulnerable and marginalized individuals in the community. For many, addiction is only one part of a complex set of health problems – problems made worse by mental illness, poverty, chronic disease and homelessness.

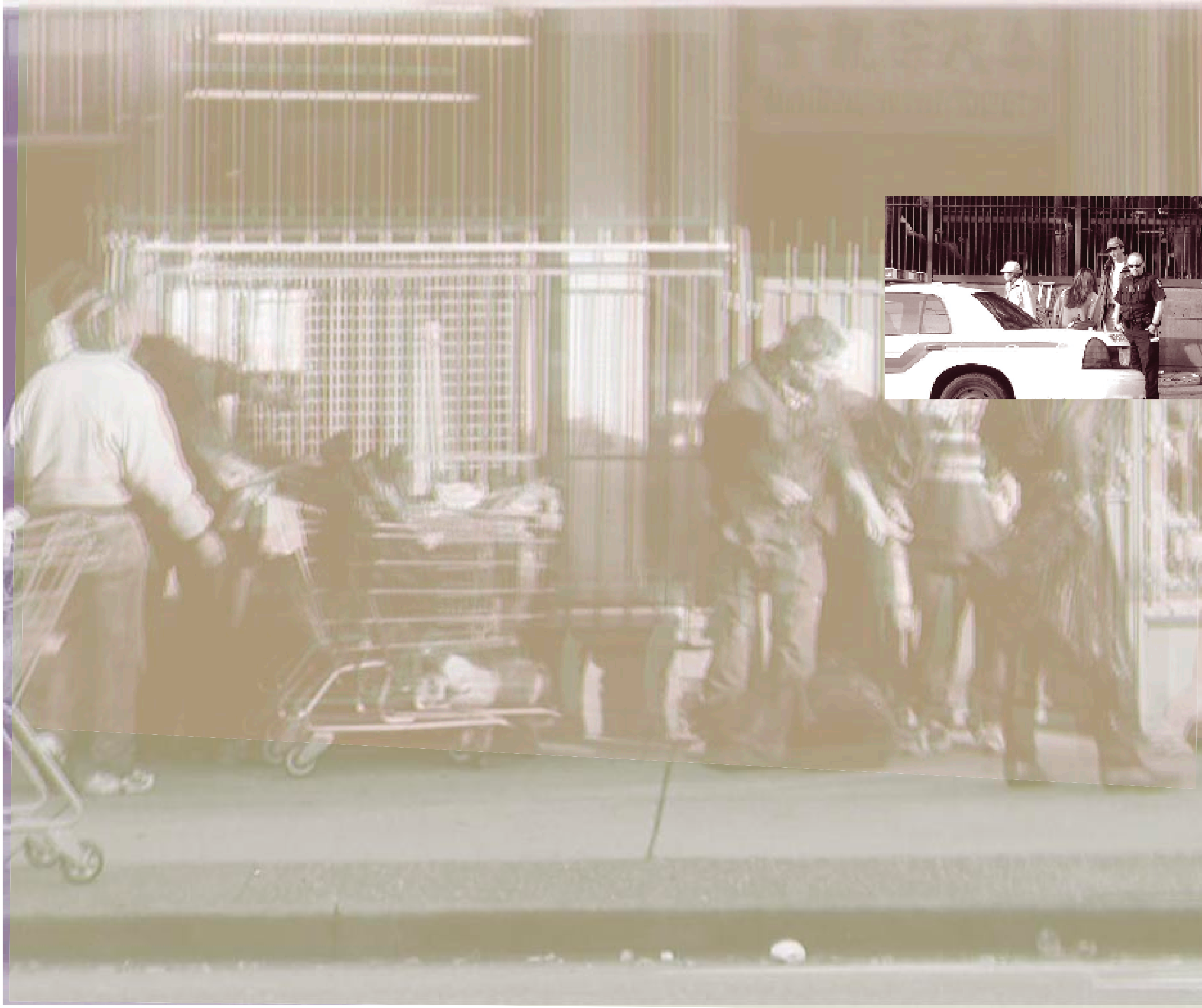

Estimates indicate there are more than 12,000 injection drug users living in Vancouver, and at least one in three live in the Downtown Eastside – the poorest neighbourhood in the city. For injection drug users living in the Downtown Eastside, more than nine out of 10 have Hepatitis C, and three in 10 are HIV positive – a rate that is 38 times the provincial average. Overdose deaths are not uncommon, and the overall mortality rate for this population is 14 times that of other BC residents.

Of the Downtown Eastside population, 1250 people live in substandard single room hotels; 650 people rely on shelters; and 200 are homeless.

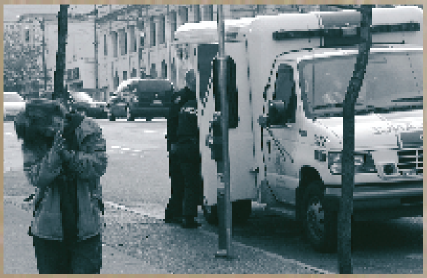

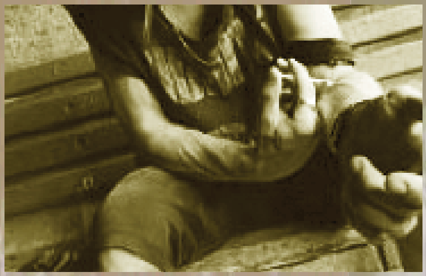

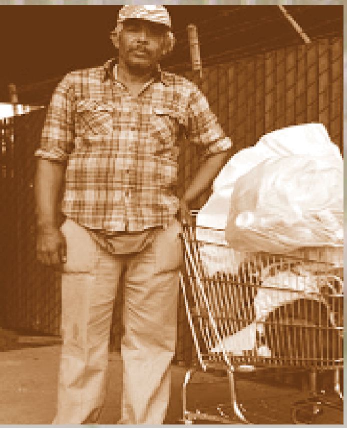

Addressing the need

Community residents, drug user groups and activists called for an innovative response to the high rates of infectious disease and overdose death. Vancouver Coastal Health, in partnership with the Portland Hotel Society, responded with an innovative program – North America's first Supervised Injection Site. Called ***in*site**, it is a safe, clean place where people with addictions can go to inject drugs and connect with health care and addiction services. The partners were supported by the Vancouver Police Department, City of Vancouver, Province of British Columbia, Injection Drug Users, community groups, academic institutions and others. Collectively, they believed the Supervised Injection Site would have the ability to positively impact people's lives.

***in*site** has been in operation since September 12, 2003 and was specifically designed to be accessible to vulnerable populations of injection drug users – men and women who use more than one drug; have both an addiction and a mental illness; have a history of trauma; have sought treatment but been unsuccessful and relapsed; live on income assistance; are Aboriginal; live in substandard housing or are homeless.

Evaluating Success

In addition to being the first Supervised Injection Site in North America, ***in*site** is also the first and only site to undergo an arms length, rigorous scientific evaluation, with all research results published in peer-reviewed journals. The British Columbia Centre for Excellence in HIV/AIDS received the contract to conduct the scientific evaluation of ***in*site**, and has been evaluating the facility in terms of meeting the following objectives:

- Increasing access to health and addiction care
- Reducing overdose fatalities
- Reducing the transmission of blood-borne infections like HIV and Hepatitis C
- Reducing injection-related infections
- Improving public order

The remainder of this report draws on the evaluation results.

INSITE SERVICES

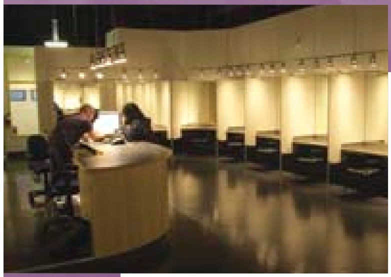

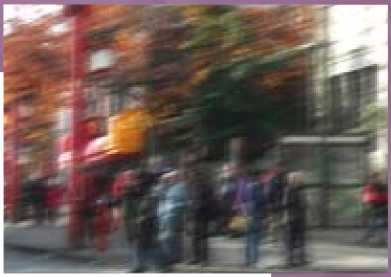

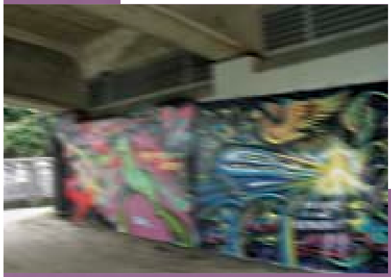

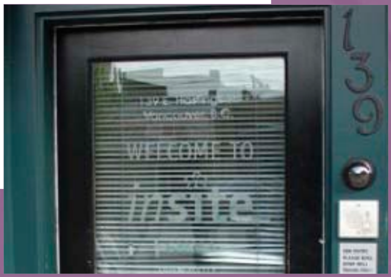

- Staffed by a combination of clinical and non-clinical staff, including peers, program assistants, RNs, Alcohol and Drug Counsellors, coordinators
- Open 18/7: 10 a.m. to 4 a.m.
- Capacity of 850 injections/day
- 12-seat injection room
- Drugs not provided on site
- Supervision of injections, including emergency response to overdoses
- Assessments, immunization and wound care
- Injection-related first aid
- Referrals to addiction treatment and other health services
- Harm reduction instruction
- Access to sterile injection equipment
- Post injection space for observation and peer interaction

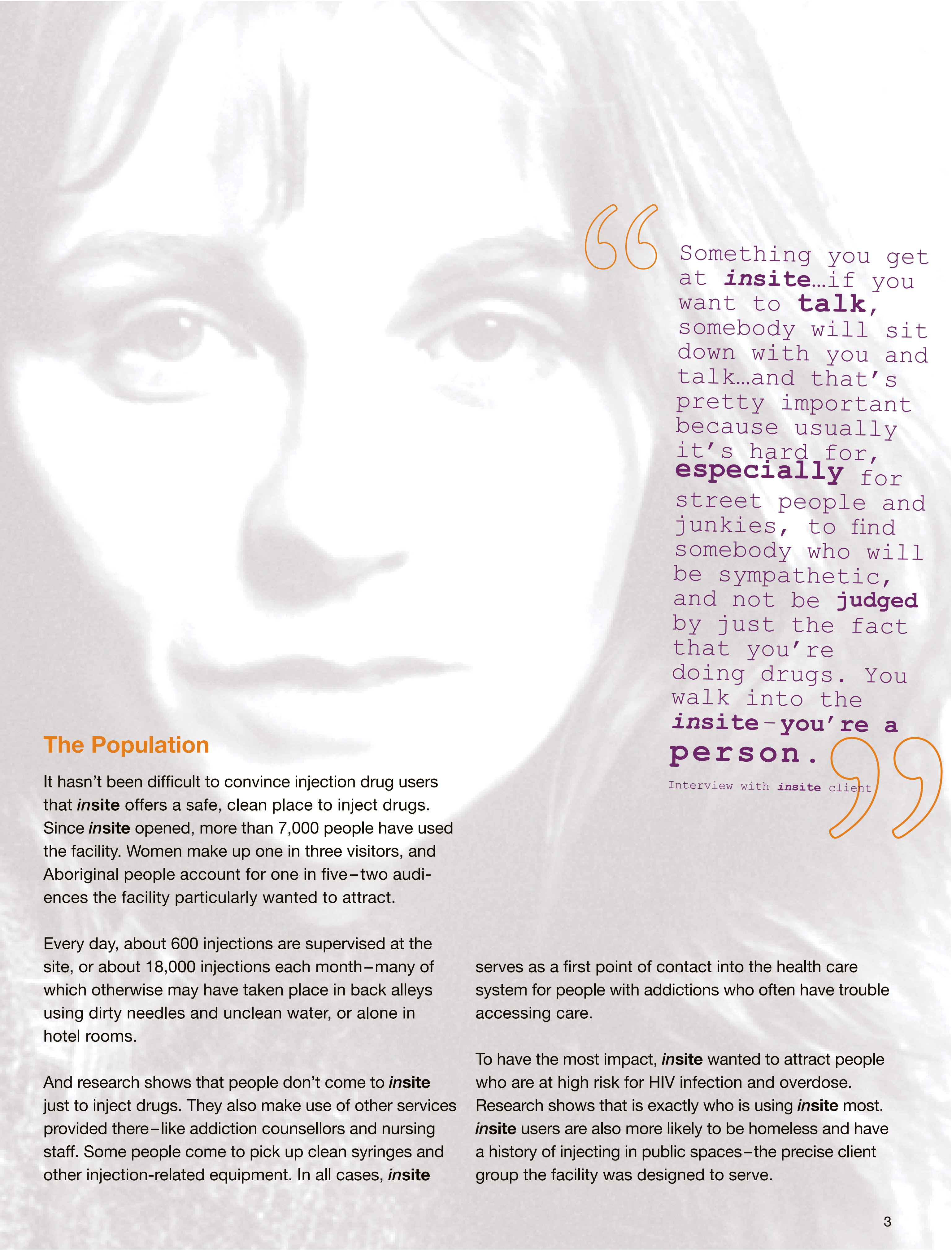

> Something you get at ***insite***…if you want to **talk**, somebody will sit down with you and talk…and that’s pretty important because usually it’s hard for, **especially** for street people and junkies, to find somebody who will be sympathetic, and not be **judged** by just the fact that you’re doing drugs. You walk into the ***insite***–**you’re a person.**
>
> Interview with ***insite*** client

The Population

It hasn’t been difficult to convince injection drug users that ***insite*** offers a safe, clean place to inject drugs. Since ***insite*** opened, more than 7,000 people have used the facility. Women make up one in three visitors, and Aboriginal people account for one in five–two audiences the facility particularly wanted to attract.

Every day, about 600 injections are supervised at the site, or about 18,000 injections each month–many of which otherwise may have taken place in back alleys using dirty needles and unclean water, or alone in hotel rooms.

And research shows that people don’t come to ***insite*** just to inject drugs. They also make use of other services provided there–like addiction counsellors and nursing staff. Some people come to pick up clean syringes and other injection-related equipment. In all cases, ***insite*** serves as a first point of contact into the health care system for people with addictions who often have trouble accessing care.

To have the most impact, ***insite*** wanted to attract people who are at high risk for HIV infection and overdose. Research shows that is exactly who is using ***insite*** most. ***insite*** users are also more likely to be homeless and have a history of injecting in public spaces–the precise client group the facility was designed to serve.

Improving Access to Care

Drug users who live on the margins may have difficulty accessing health care. And without this care, the chances of improving health outcomes are slim. By visiting ***in*site** and being exposed to health care professionals in a non-threatening environment, many clients willingly ask for referrals to other health and social services. Over a one-year period, ***in*site** made more than 2,000 referrals to other services, with about 40 percent of the referrals to addiction counseling.

People using ***in*site** are more likely to enter withdrawal management (detox) programs, and people using ***in*site** who also talk with addiction counselors are even more likely to enter detox. In fact, one in five regular visitors to ***in*site** began a detox program, showing that ***in*site** is a proven entry-point for the Downtown Eastside's highest-risk injection drug users. Detox programs are an important marker of addiction treatment system use, as most addiction treatment programs first require people to complete a detox program.

Researchers also compared the rate of detox program use among people using ***in*site** – before and after ***in*site** opened – with the rate of detox use going up substantially after people had been using the facility.

Saving Lives

Research results show that ***in*site** is playing an important role in managing overdoses that occur at the site – overdoses that would, in all likelihood, be occurring on the streets if the facility didn't exist, and could result in death.

Overdoses have been common at ***in*site**, with almost 500 occurring over a two-year period, but none resulted in a fatality. In fact, ***in*site** staff managed the majority of overdoses on their own, with only four in 10 needing ambulance support, and fewer than one in 10 resulting in a transfer to hospital.

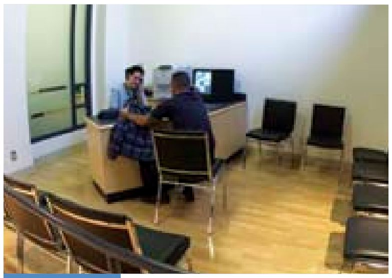
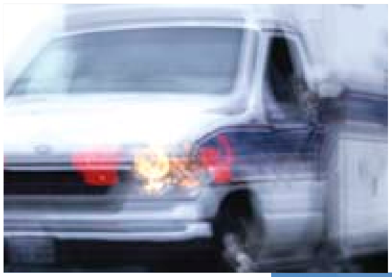
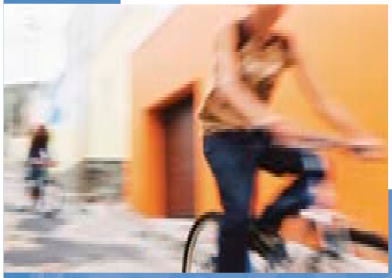
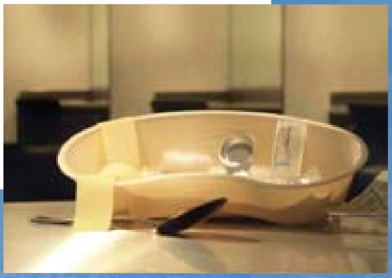

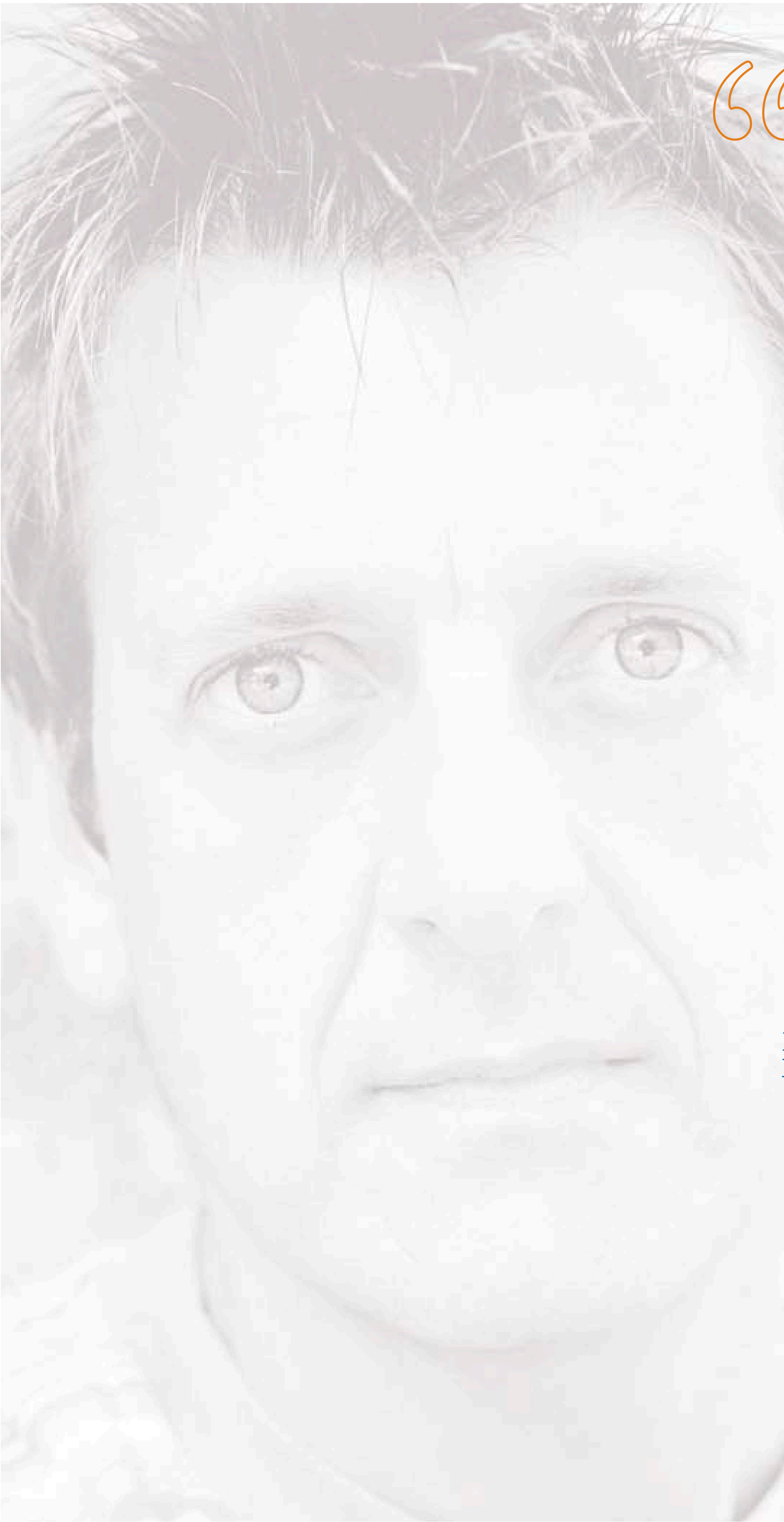

"It was about **11 o'clock at night** and I had gone in and I actually had gotten a huge amount of heroin…five papers. I had gone there and I decided to do it **all in one**… And it was like, lights out…I come to–I'm laying on my back and I don't even know where I'm at. I'm **very scared**…I just remember seeing the ***insite*** lady talking to me, **holding my hand**, telling me that **everything was gonna be okay**. There was an officer there, and there was a paramedic there. If I had gone home and did that…I probably wouldn't be here, cause no one was at home. I was by myself, so I definitely would have **overdosed** and I probably would have ended up killing myself."

Interview with ***insite*** client

Improving Health

Hepatitis C and HIV rates in the Downtown Eastside parallel those of developing countries. A key goal of ***insite*** is to reduce the high rates of transmission of infectious diseases. Sharing syringes used to inject drugs is a ready source of transmission, so supplying a safe, clean place, and making sterile equipment available, is one way to stop the spread of disease.

Research studies show that people using ***insite*** are 70 percent less likely to share syringes than injection drug users who do not use the facility or use it infrequently. And rates of syringe sharing in the community are lower than they were in the past, indicating ***insite*** has had an impact on behaviour well beyond the walls of the facility.

For injection drug users, soft tissue infections from injecting is the most frequent reason for hospitalizations, including visits to the emergency department, as well as overnight stays. Many infections are the result of injecting with unclean equipment, not cleaning the skin first, or using dirty water. One of the services ***insite*** offers clients is education on how to safely inject – with at least one in three people visiting ***insite*** receiving this education.

And the education is working. Research shows that people using ***insite*** substantially increase their use of sterile injecting practices and decrease injecting in public – the latter a practice known to increase the risk for infectious disease transmission.

Impact on the Community

When ***insite*** was still in the conception stage, some people worried that a Supervised Injection Site would send the wrong message about drug use and prevent people from stopping using. Others thought ***insite*** would make injecting drugs more attractive. Some people still believe both of these to be true.

But the research shows these concerns are unwarranted, because drug use patterns in the community have not worsened as result of opening ***insite***. And crimes related to drug use have not increased.

IMPACT

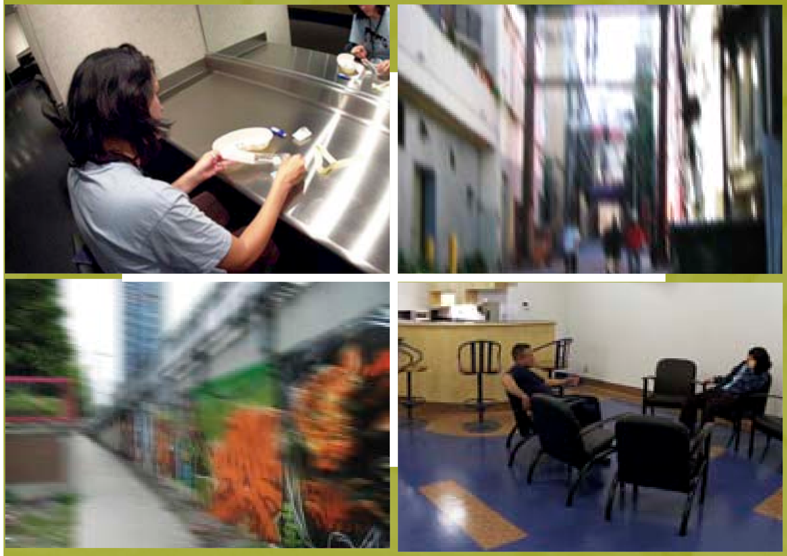

- Reaching high-risk injection drug users
- Decreasing public injection
- Reducing needle sharing
- Reducing HIV risk behaviour
- Increasing use of addiction treatment
- Increasing referral to community services
- Reducing bacterial infections
- Not increasing crime, public disorder, drug dealing, litter or relapse rates

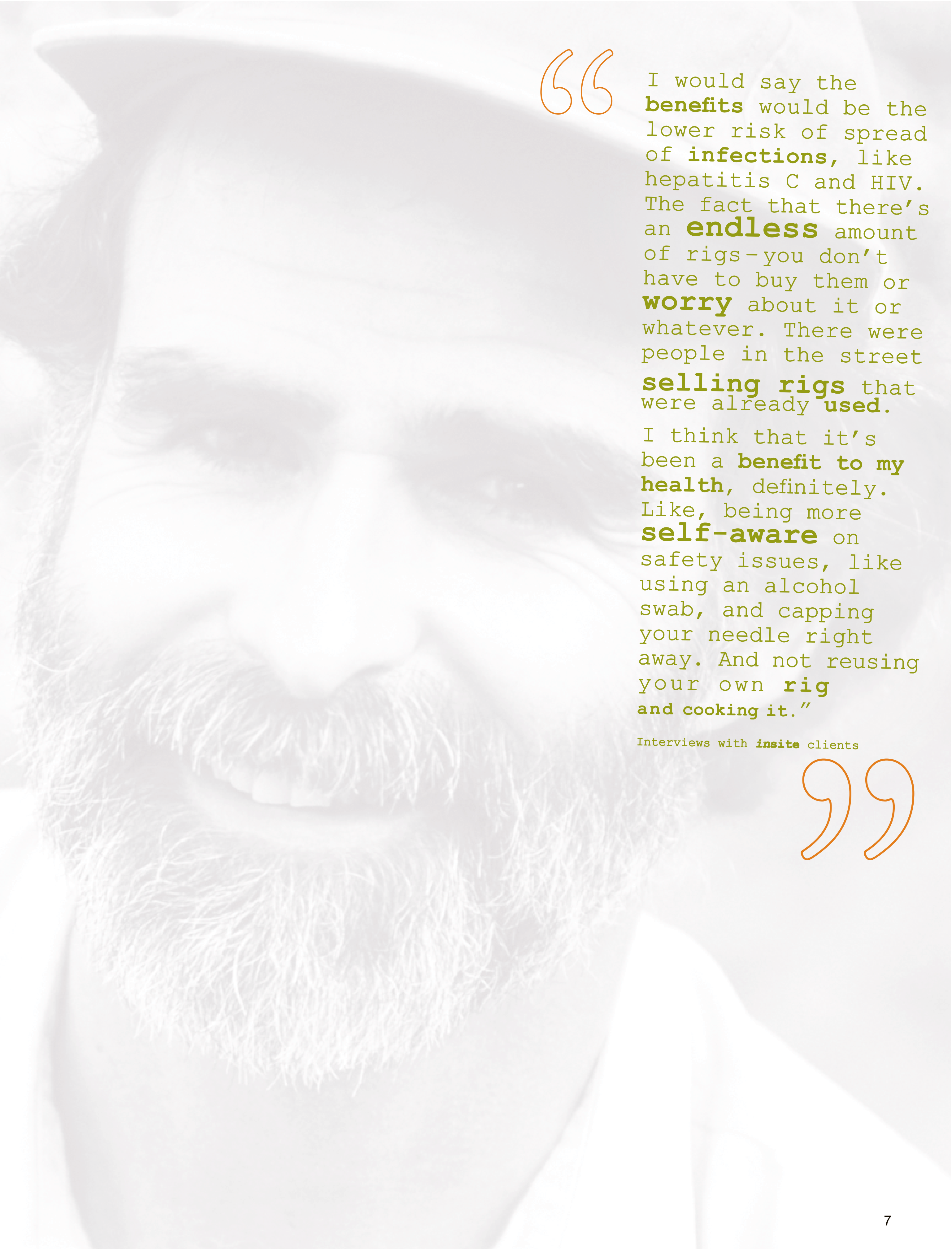

"I would say the **benefits** would be the lower risk of spread of **infections**, like hepatitis C and HIV. The fact that there's an **endless** amount of rigs – you don't have to buy them or **worry** about it or whatever. There were people in the street **selling rigs** that were already **used**.

I think that it's been a **benefit to my health**, definitely. Like, being more **self-aware** on safety issues, like using an alcohol swab, and capping your needle right away. And not reusing your own **rig** **and cooking it**."

Interviews with ***insite*** clients

Creating a Safer Neighbourhood

While **insite** was developed to reduce drug related harms and improve people's health, it was also created to help respond to the growing problem of public disorder related to injection drug use in the Downtown Eastside. Prior to **insite** opening, researchers collected data on how much public injecting was taking place in the neighbourhood. They then collected the same information weeks after **insite** opened and found that the opening of **insite** resulted in substantial reductions in public disorder, including a reduction in the number of people injecting in public and the number of discarded syringes found on city streets.

Looking Forward

Scientific research studies prove that **insite** is making a real difference in the lives and health of people who use the facility. And it is meeting community needs for improvements in public order. The people who could benefit most from using **insite** are some of its most frequent visitors. Needle sharing, one of the highest risk factors for HIV and hepatitis C infection, has been dramatically reduced. And the education on safe injecting **insite** clients learn, they also practice when they are away from the facility.

Harms related to injection drug use are also on the decline – from transmission of infectious diseases to improved wound care; from more people accessing the health care system to more people with addictions accessing treatment.

Most importantly, people who overdose at **insite**, unlike many who overdose on the street or alone in a hotel room, live to tell the story.

Researchers from the BC Centre for Excellence in HIV/AIDS conclude that "**insite** is a highly innovative initiative that greatly complements existing services by providing a safer place for injection of illicit drugs. It also provides a place to engage high-risk injection drug users, especially those who are not well connected to the health care system."

For people with addictions, **insite** is proving to play an essential role as an entry point to health care services, including addiction treatment, and has become a vital part of the health care continuum. **insite** is decreasing drug-related harms, reducing the risk of transmission of infectious disease, reducing the incidence of injection-related infections, and most importantly – saving lives.

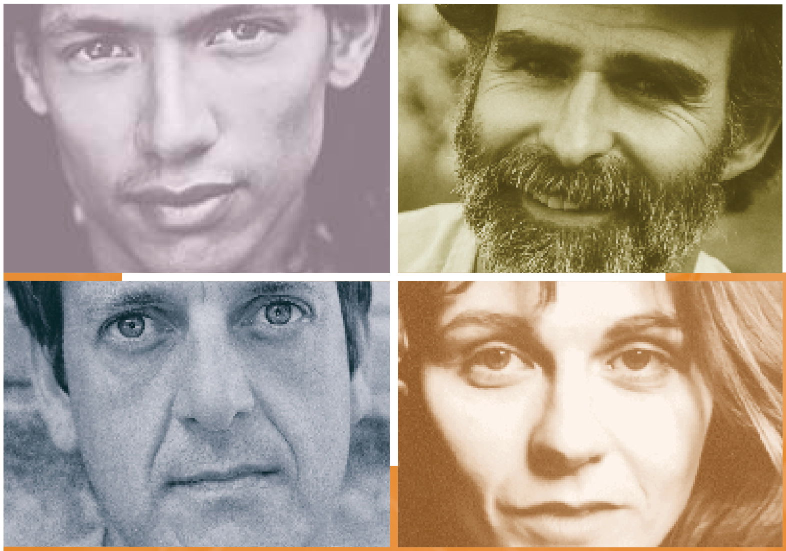

INSITE BY THE NUMBERS

From April 1, 2004 to March 31, 2006

7,278 unique individuals registered at *in***site** • Women made up 26% of clients • Aboriginal people made up 18% of clients • Heroin was used in 41% of injections • Cocaine was used in 27% of injections • Morphine was used in 12% of injections • 453 overdoses resulted in no fatalities • 4,084 referrals were made with 40% of them made to addiction counselling • Referral to withdrawal management: 368 • Referral to methadone maintenance: 2 per week • Daily average visits: 607 • Average number of visits per month, per person: 11 • Busiest day: May 25, 2005 – 933 visits in 18 hours • 6,227 – number of nursing care interventions • 2,055 number of nursing interventions for abscess care

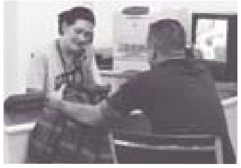
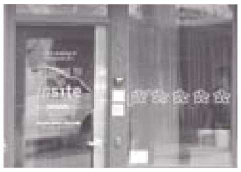
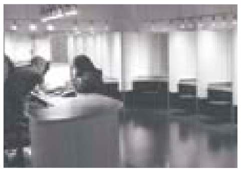
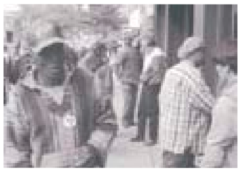
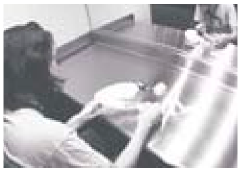
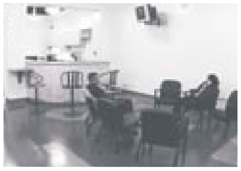

SIS PARTNERS

Community Members

Vancouver Coastal Health

PHS Community Service Society

Vancouver Police Department

City of Vancouver

Office of the Provincial Health Officer

BC Centre for Excellence in HIV/AIDS

University of British Columbia

Vancouver Agreement Partners

L.I.N.E.S.
