# Supplementary material for: Insite: Canada's landmark safe injecting program at risk
Source: Harm Reduct J. 2006 Aug 9;3:24. doi: 10.1186/1477-7517-3-24 (PMC1562403; doi:10.1186/1477-7517-3-24)
Supplement: Additional file 2 — a review which summarizes these research studies. [file 1477-7517-3-24-S2.doc]

**Vancouver’s Supervised Injection Site: Research Summary**

When launched in September 2003, Vancouver’s Supervised Injection Site (*in***site**) began providing vital health care and referral services to drug users on Vancouver’s downtown eastside. It also began a three-year research program to provide the community, government, other health care agencies and the global research community much needed data about the efficacy of such a facility on health outcomes and community safety and security.

The research has been conducted by the BC Centre for Excellence in HIV/AIDS, recognized as one of the world’s leading research organizations. The Centre’s research was subject to rigorous, independent third-party evaluation, and published in peer-reviewed journals including the New England Journal of Medicine, the British Medical Journal, the Canadian Medical Association Journal and The Lancet. Results include:

- *in***site** is leading to increased uptake into detoxification programs and addiction treatment. *(New England Journal of Medicine)*
- *in***site** has not led to an increase in drug-related crime, rates of arrest for drug trafficking, assaults and robbery were similar after the facility’s opening, and rates of vehicle break-ins/theft declined significantly. (*Substance Abuse Treatment, Prevention, and Policy)*
- *in***site** has reduced the number of people injecting in public and the amount of injection-related litter in the downtown eastside. (*Canadian Medical Association Journal*)
- *in***site** is attracting the highest-risk users – those more likely to be vulnerable to HIV infection and overdose, and who were contributing to problems of public drug use and unsafe syringe disposal. (*American Journal of Preventive Medicine*)
- *in***site** has reduced overall rates of needle sharing in the community, and among those who used the supervised injection site for some, most or all of their injections, 70% were less likely to report syringe sharing. (*The Lancet*)
- Nearly one-third of *in***site** users received information relating to safer injecting practices. Those who received help injecting from fellow injection drug users on the streets were more than twice as likely to have received safer injecting education at *in***site**. (*The International Journal of Drug Policy*)
- *in***site** is not increasing rates of relapse among former drug users, nor is it a negative influence on those seeking to stop drug use. (*British Medical Journal*)

Other research results show:

- 7,278 unique individuals registered at *in***site**
- Women made up 26% of clients
- Aboriginal people made up 18% of clients
- Heroin was used in 41% of injections
- Cocaine was used in 27% of injections
- Morphine was used in 12% of injections
- 453 overdoses resulted in no fatalities
- 4,084 referrals were made with 40% of them made to addiction counselling
- Referral to withdrawal management: 368
- Referral to methadone maintenance: 2 per week
- Daily average visits: 607
- Average number of visits per month, per person: 11
- Busiest day: May 25, 2005 (933 visits in 18 hours)
- Number of nursing care interventions: 6,227
- Number of nursing interventions for abscess care: 2,055
